# An Invited Reply to: A Comment on: Migrating silver eels return from the sea to the river of origin after a false start (2021) by Tambets M *et al.*

**DOI:** 10.1098/rsbl.2022.0429

**Published:** 2022-11-23

**Authors:** Meelis Tambets, Einar Kärgenberg, Ain Järvalt, Finn Økland, Martin Lykke Kristensen, Anders Koed, Priit Bernotas

**Affiliations:** ^1^ Wildlife Estonia, Veski 4, Tartu 51005, Estonia; ^2^ Estonian Marine Institute, University of Tartu, Vanemuise 46, Tartu 51014, Estonia; ^3^ Chair of Hydrobiology and Fishery, Institute of Agricultural and Environmental Sciences, Estonian University of Life Sciences, Kreutzwaldi 5, Tartu 51006, Estonia; ^4^ Norwegian Institute for Nature Research NINA, PO Box 5685, Trondheim No-7485, Norway; ^5^ Technical University of Denmark, National Institute for Aquatic Resources, Silkeborg 8600, Denmark

The population of European eel (*Anguilla anguilla*) has been declining extensively and needs efficient conservation measures. Relevant information on the biology of this species (including spawning migration) is necessary for management decisions. We discovered an unprecedented behavioural pattern—after migrating to the Baltic Sea, 21% of the silver eels, tagged with acoustic transmitters, returned to the Narva River [1]. After that, they migrated to the sea again and started their migration towards their spawning area.

Our research prompted Rohtla *et al.* to write a Comment [2]. In our opinion, this Comment contains misunderstandings that we consider necessary to point out in this Reply. We do see some useful and valid elements in their Comment, but also speculation and untrue statements misleading readers. A striking example of the latter is included in the opening paragraph of their text [2]. One of the important criteria for any study is its novelty. They make a claim that tries to diminish the novelty of our study [1]. The statement that ‘similar movements to freshwater have been observed in silver stage American eels (*Anguilla rostrata)*’ has been shown in the studies of Jessop *et al*. [3,4], is incorrect. In our article, we show that the fish that descended to the sea as silver eels returned to the river. Jessop *et al*. discuss the movement of fish from the sea to the river before they silver and begin the spawning migration. The studies of Jessop *et al.* [3,4] clearly indicate that the otoliths of some silver eels caught in freshwater during downstream migration show inter-habitat migration as yellow eels, which is also confirmed by Jessop (B. M. Jessop 2022, personal communication). They do not show that silver eels that have departed freshwater have returned for another period of freshwater or estuarine residence. They also do not show silver eels moving from estuary to freshwater—they do show younger, presumably yellow, eels moving from estuary to freshwater and vice versa. It has not been observed that some silver eels change direction in the estuary and return to the river for renewed migration the next year. This is a very important difference. Rohtla *et al.* misunderstood the text of Jessop *et al*. and conveys a misinterpretation to the reader.

Another consequence of the misinterpretation of the articles of Jessop *et al.* is that Rohtla *et al.* [2] offer the *farewell visits* hypothesis, described by Jessop *et al.* [4], in explaining the circumstances of the phenomenon of return migration to the river. The term *farewell visit* refers to the movement of yellow eels between the sea and the river where silvering has not been previously demonstrated. In our study [1], we consider the case where some of the silver eels that have descended to the sea immediately migrate towards the exit of the Baltic Sea, while some come back to the river and make a new start towards the sea. For those fish that returned to the river, the first start to the sea was unsuccessful, they did not continue with a longer migration, and they made a new start. We called this unsuccessful start *false start* and we think it is an appropriate expression in such a context.

Rohtla *et al.* [2] are right that the reasons for the return are not yet well known. We also state this in our article [1]. We analysed the factors affecting migration that could be addressed (total length, total weight, Fulton index, Durif index, Pankhurst index, water temperature and discharge, moon phase). We are open to a fact-based discussion about what causes the observed behaviour.

Rohtla *et al.* [2] begin to speculate what factors could have caused such behaviour, pointing out very different variants (long-ago or recent translocation of fish, too complex and long freshwater system for navigation, possible injuries, low silvering rate, possible reversion of silvering characteristics, need to reorient the fish's internal magnetic compass). There are many more possible explanations. We suggest that the underlying reasons for the observed behaviour should be investigated in future studies.

Rohtla *et al.* [2] describe the beginning of eels' movements as ‘it is likely that many eels just descended with the flow’. This interpretation is very likely inadequate. First, to avoid passive descent with flow, we avoided the release of the fish in the channel with current, and therefore we chose the release site in the reservoir with stagnant water. When analysing the start times of eels' downstream migrations, one should conclude that these were active movements and not downstream migration. Also, fish moved in other directions before migrating downstream.

Some of the opinions of Rohtla *et al.* [2] seem implausible in light of the known facts. For example, they claim that ‘Tambets *et al*. [1] did not determine eel developmental stage at the time when they returned to the river up to a year after descending to the sea, it cannot be claimed that the eels returning to the river were indeed silver eels'. If possible, we would have determined the developmental stage of the fish returning to the river, but the return of fish was not expected and thus we did not make recapture efforts. Therefore, one can only speculate about the condition of the returning eels. Considering the fact that the fish that returned to the river migrated to the Danish straits in the winter of 2019–2020 and two of them returned to the river in the last half of October 2019, we find it highly unlikely that they returned as yellow eels. It is probable that the fish that migrate in the fall and winter are already silvered at the end of October of the same year. The suggestion of Rohtla *et al.* that these eels could have been yellow is incomprehensible.

In our opinion, Rohtla *et al.*'s Comment [2] misses the main message of our article. It is the discovery that under certain conditions, for whatever reason, an eel can behave in the manner described in our article—return to the river after descending to the sea as silver eels, and then descend to the sea again and begin the spawning migration. Their final conclusion repeats the statement in our article that ‘continued research on this phenomenon on stocked and naturally invading eels is needed’.

As previously mentioned, we still do not know what caused the eels to exhibit this interesting behaviour. Water systems similar to our study system—complex river systems with impounded and stagnant sections with migration obstacles—are numerous around the world, and the eel stocking and translocation method has been widely used for decades. This behaviour pattern, which is the subject of discussion at the moment, may be characteristic of eels more generally.

## Data Availability

This article has no additional data.
